# An eye-tracking study of letter-sound correspondence in Japanese-speaking 2- to 3-year-old toddlers

**DOI:** 10.1038/s41598-020-79062-y

**Published:** 2021-01-18

**Authors:** Hiroki Higuchi, Yuko Okumura, Tessei Kobayashi

**Affiliations:** Innovative Communication Laboratory, NTT Communication Science Laboratories, 2-4, Hikaridai, Seika-cho, Kyoto, 619-0237 Japan

**Keywords:** Psychology, Human behaviour

## Abstract

Although the acquisition of letter-sound correspondences is a critical step in reading development, how and when children develop such correspondence remains relatively unexplored. In this study, we focused on Japanese hiragana letters to examine the implicit letter-sound correspondence using an eye-tracking technique for 80 Japanese-speaking toddlers. The results showed that 32- to 48-month-olds (but not 24- to 32-month-olds) directed their gaze at the target letter. An additional experiment on a letter-reading task showed that 32- to 40-month-olds could barely read the presented hiragana letters. These findings suggest that toddlers have already begun to grasp implicit letter-sound correspondences well before actually acquiring the ability to read letters.

## Introduction

The acquisition of word reading skills is a critical landmark to become a fluent reader. Dual route models of reading suggest that word reading entails (a) a non-lexical route that serially converts letters (graphemes) into correspondent sounds and (b) a lexical route that converts words (orthography) into sounds^1^. Reading by the non-lexical route generally needs more time to process long words than short words because this route serially converts each grapheme into corresponding sounds. In contrast, reading by the lexical route converts known, written words of any length into sounds quite promptly because it retains a direct connection between a representation of a written word and a sound. However, it cannot deal with unknown written words. This suggests that literate adults can fluently read words using both types of outputs from lexical routes for known word reading and from non-lexical routes for unknown word reading.

How children develop these routes is a critical issue for identifying their reading development and the underlying mechanisms of developmental dyslexia. Previous studies have shown that English-speaking children^2,3^ as well as Italian- and Japanese-speaking children^4,5^ cultivate a non-lexical route at the earlier stages of development and subsequently a lexical route. Theoretically, the self-teaching hypothesis^3^ argues that successful decoding using non-lexical information provides a favorable opportunity for developing direct connections between words (orthography) and sounds. Since children read words that they do not actually know how to read, they need to rely on grapheme-to-sound correspondence rules in the non-lexical route. In the case of letters (graphemes) that are mapped onto multiple sounds, children can discern correct sounds from multiple candidates in the rules of letter-sound correspondence (e.g., /hIt/, /hiːt/, /hiːl /, or /hæt/ in ‘heat’) by looking up the spoken word lexicons. Since children develop spoken words before reading words, correct pronunciation (i.e., /hiːt/) might be strongly activated without any external teaching signals. Indeed, a longitudinal study of early reading skills concluded that knowledge, including letter sounds at kindergarten, predicts later reading-related skills^6^.

The remaining question is when and how children develop a letter-sound correspondence, which is a critical factor for non-lexical processing. One factor that contributes to alphabetic letter-sound correspondence acquisition is letter-naming knowledge^6^. Another factor is phonological awareness, which refers to the ability to decompose a spoken word into a minimal sound unit (i.e., a phoneme in English) and manipulate it. Since poorer phonological awareness is related to developmental dyslexia^7^, great attention has been focused on the contribution of phonological awareness to reading acquisition. For example, English-speaking 5-year-olds showed a strong correlation between phonological awareness in phoneme isolation and letter-sound naming performance (letter-sound correspondences)^8^. One possible interpretation of this correlation is that phonological awareness facilitates letter-sound correspondence because the extraction of sound units (phonemes) simplifies the construction of the connections between letters (grapheme) and sounds. Indeed, phonological awareness showed a significant contribution to letter-sound knowledge in English-speaking kindergarteners^9^. However, a recent study proposed a contrary suggestion that a letter-sound correspondence facilitates phonological awareness^10^. These studies suggest a bidirectional relationship between letter-sound correspondences and phonological awareness. However, how children develop both abilities in their interaction with each other is less well understood.

Identifying the developmental interaction between letter-sound correspondences and phonological awareness is important for better understanding of both abilities. For phonological recognition, children can implicitly recognize their native language sounds (phonological perception) before manipulating them (phonological awareness). For example, English-speaking 6-month-olds can recognize vowel sounds^11^, and English-speaking 12-month-olds can perceive consonant sounds^12^, indicating that infants by 12 months of age have already acquired the phonological system of their own language. In contrast, the emergent development of letter-sound correspondences is less understood. Previous studies mainly assessed explicit letter-sound correspondences in which children are requested to name letters; its emergent development has been neglected. Considering oral language development, English-speaking 14-month-olds can rapidly form a correspondence between spoken nonsense words and object referents before producing their first words^13^. Likewise, children might understand letter-sound correspondences before beginning to identify letters. Previous studies have shown that young children have already acquired the fundamental abilities to perform such letter-sound correspondences as phonological perception^11^, visual statistical learning^14^, and cross-modal matching^13^.

Additionally, toddlers might be implicitly exposed to letters from their environments at earlier stages of language development. A possible source of letter input is from picture books. A previous study reported a significant correlation between the acquisition order of hiragana in 4- to 5-year-olds and their frequency in picture books^15^, suggesting a relationship between picture-book reading and the acquisition of letter-sound correspondences. Other studies reported that 9-month-olds begin to perform shared-book reading^16^, implying early exposure to print in picture books.

Given previous evidence that toddlers by their third birthday have already acquired the fundamental abilities to perform letter-sound correspondences and have a great deal of experience seeing letters in their environment (including picture books), they might *implicitly* learn letter-sound correspondences earlier than previously expected. Here we investigate whether Japanese-speaking toddlers can understand implicit letter-sound correspondences before they learn to read their letters. The Japanese language system consists of syllabic kana (hiragana and katakana) and logographic kanji. Japanese kanji characters, which are comprised of thousands of characters, are visually more complex than kana letters. The acquisition of kanji reading generally comes after hiragana. We focus on Japanese hiragana for two reasons. First, it enables us to exclusively assess letter-sound correspondences because there is no confliction between letter names and letter sounds. In English, letter names are often different from letter sounds, which may confuse children when performing letter-sound tasks. Second, most Japanese-speaking children attend preschool. Although a previous study showed the contribution of preschool education for emergent literacy levels^17^, over 90% of Japanese 4- and 5-year-olds attend preschool or kindergarten^18^.

To evaluate toddlers' understanding of implicit letter-sound correspondences in Japanese hiragana, we used an intermodal, preferential-looking paradigm based on an eye-tracking technique^19–21^. This paradigm is often used to measure early syntax and lexical comprehension. In this method, a pair of visual stimuli is presented side by side, and a target stimulus is auditorily presented. Fixation to the target visual stimuli is used as an index for understanding the referents. We applied this method to measure the toddlers’ letter-sound correspondences, such that two hiragana letters were presented side by side on the screen, and the corresponding sounds of the target letters were auditorily presented. If toddlers understand the letter-sound correspondences, we expect them to spend more time fixating on the target visual stimuli. Since previous studies showed that Japanese-speaking children start to learn hiragana letter-sounds around 3 to 4 years of age^22^, we evaluated the implicit letter-sound correspondences for toddlers at from 24 to 48 months. To examine their developmental changes, we divided them into three age groups: young (24–32 months), middle (32–40 months), and old (40–48 months). The toddlers’ reading attainment was verified by a letter-reading task. We also checked the parent’s recognition of their toddlers’ acquisition of hiragana-letter reading by questionnaires. Additionally, their parents completed a vocabulary checklist (Japanese version of the MacArthur-Bates Communicative Development Inventories: CDI). Based on these measures, we investigated hiragana letter-sound correspondences before acquiring the ability to read letters.

## Results

### Hiragana letter-sound correspondences

**Figure 1:**
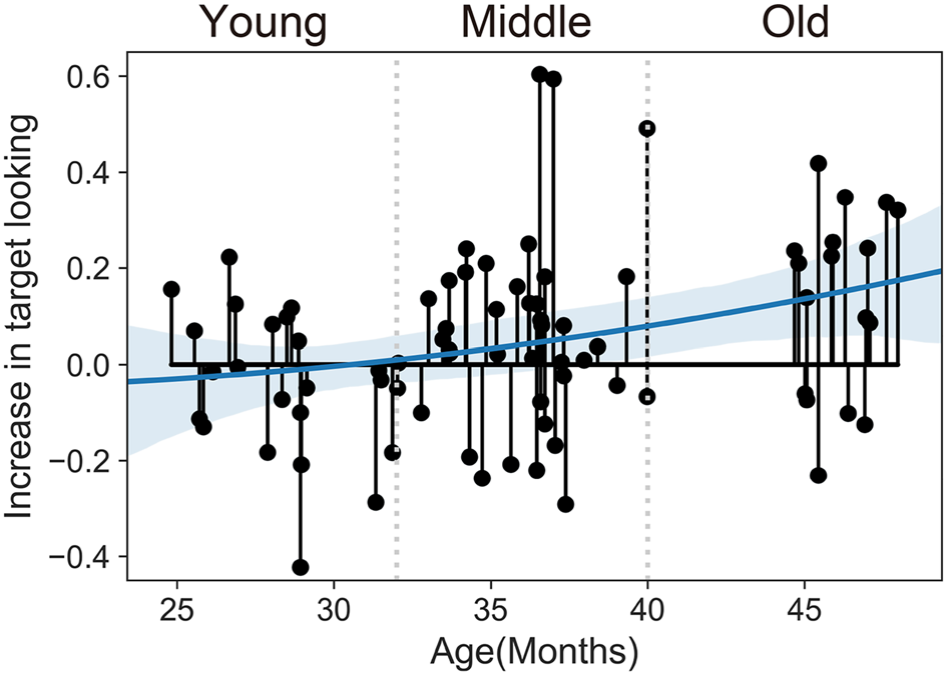
Subject mean values in target-looking increase for three age groups. All data were calculated over 367- to 4000-ms time windows. Toddlers in the young group (24–32 months) did not significantly fixate on sound-matched hiragana letters. However, index for toddlers in the middle (32–40 months) and the old (40–48 months) groups significantly exceeded zero. The blue line indicated polynomial regression line (order = 2) with 95% confidence interval (light blue).

First, we showed that the toddlers’ target looking tended to gradually increase with age using polynomial regression (Fig. 1). Next, we evaluated whether the toddlers of each age group showed significantly larger values than zero at each age range by one-tailed Wilcoxon test. While the toddlers in the young group (24–32 months) did not show significantly larger values than zero (Fig. 1, *M* = − 0.04, *p* = 0.85, Wilcoxon test), those in the middle group (32–40 months) did (Fig. 1, *M* = 0.06, *p* = 0.03, Wilcoxon test). The toddlers in the old group (40–48 months) also showed significantly larger values than zero (Fig. 1, *M* = 0.13, *p* < 0.01, Wilcoxon test). These results suggest that the toddlers in the middle and old groups (but not the young group) correctly performed hiragana letter-sound correspondences in the eye-tracking task. The results for each item-pair are shown in the supplementary material.

An additional analysis of the correlation showed no significant relation between the scores of the target-looking increase and the productive vocabulary sizes measured by CDI (*r* = 0.07, *p* = 0.64), suggesting that vocabulary development is less likely to affect the acquisition of letter-sound correspondences.

### Hiragana-reading accuracy

**Figure 2:**
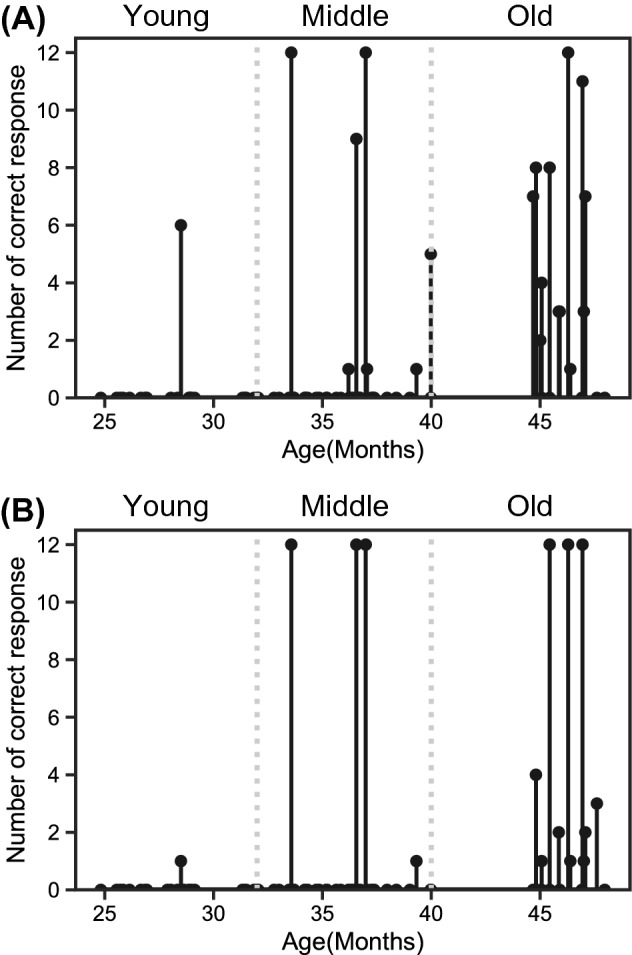
Toddlers’ reading attainment evaluated by (**A**) an experimenter and (**B**) their parents for toddlers in young (24–32 months), middle (32–40 months) and old (40–48 months) groups.

After the eye-tracking experiment, the experimenter checked their hiragana-reading accuracy for 12 letters (Fig. 2A). The toddlers in the young group could not read most of the letters (*M* = 0.3 ± 1.3 letters, range = 0–6 letters). Likewise, those in the middle group could not read most of them (*M* = 1.0 ± 3.0 letters, range = 0–12 letters). The reading accuracy for the toddlers in the old group varied across participants, although they could read some of them (*M* = 4.1 ± 4.0 letters, range = 0–12 letters).

### Hiragana-reading accuracy evaluated by their parents

Before the eye-tracking experiment, we checked the parent’s recognition of their toddlers’ acquisition of hiragana-letter reading for 12 letters (Fig. 2B). The parents in the young group reported that their children could not read most of the letters (*M* = 0.0 ± 0.2 letters, range = 0–1 letters), and those in the middle and old group also reported that their toddlers could hardly read any hiragana (*M* = 0.9 ± 3.1 letters, range = 0–12 letters; *M* = 2.9 ± 4.5 letters, range = 0–12 letters).

## Discussion

This study focused on Japanese hiragana and investigated whether toddlers can understand hiragana letter-sound correspondences before they can read the letters themselves. An eye-tracking experiment revealed that the toddlers aged 32–40 months tended to look significantly longer at sound-matched letters than sound-non-matched letters. Nevertheless, these toddlers cannot read most of the letters, suggesting that toddlers who cannot read hiragana letters yet start to understand implicit letter-sound correspondences.

Although studies that investigate letter-sound correspondences in toddlers before 3 years of age are limited not only in Japanese but also in alphabetic scripts, a previous eye-tracking study on letter-sound knowledge showed that English-speaking 10- to 18-month-olds failed to find fixation toward sound-matched target letters^23^. Their findings are consistent with the failure to fixate on sound-matched hiragana letters in the younger 24- to 32-month-olds of our present experiment. Therefore, it is reasonable to assume that toddlers begin to acquire the ability to perform implicit letter-sound correspondences around their third birthday. However, it remains unclear whether the onset of implicit letter-sound correspondence is universal across languages. The rules of letter-sound correspondences differ greatly by language. The Japanese hiragana writing system is quite transparent since each hiragana letter is mapped onto just one sound. In contrast, English letters or graphemes cannot simply correspond to one sound. Based on the differences in writing systems, the acquisition of letter-sound correspondence in Japanese hiragana is probably easier than in opaque scripts like English^24^. In other words, Japanese-speaking toddlers who learn a transparent system like hiragana may acquire letter-sound correspondences earlier than those who learn an opaque orthography.

As noted in our introduction, one critical issue regarding the development of letter-sound correspondences is the interaction between letter-sound correspondences and phonological awareness during the early stages of literacy acquisition. Although the present study showed that toddlers around their third birthday already understand implicit letter–sound correspondences, a previous study reported the emergence of phonological awareness around 4 and half years of age^25^. This implies that implicit letter-sound correspondence helps acquire phonological awareness. However, recent studies reported that toddlers develop fundamental phonological processing (i.e., phonological perception) much earlier. For example, 24-month-olds showed phonological priming^26^ and the phonological neighborhood effect^27^, implying that toddlers begin to form phonological lexicon around 2 years of age. Therefore, it is reasonable to assume that the implicit knowledge of letter-sound correspondences and phonological perceptual ability reciprocally boost the development of each other, and subsequently that toddlers acquire explicit letter-sound correspondences and phonological awareness.

Another factor that enables toddlers to learn letter-sound correspondences is the formation of visual letter representation through visual exposure to them. Studies assessing the development of visual object recognition showed that toddlers develop the ability to abstract visual objects from 17 to 24 months^28,29^. They need to recognize letters invariantly due to size and font changes and form abstract representations of them. Therefore, the emergence of the ability to abstract visual objects may help them acquire letter-sound correspondences.

This study has some limitations. First, we have only shown toddlers’ letter-sound correspondence in Japanese yet. Unlike English writing systems, there is no distinction between letter names and sounds in Japanese kana-writing systems (i.e., hiragana and katakana). For instance, Japanese hiragana letter ‘ ’ is called /a/ and also sounds like /a/ in words. Thus, the present results, which we interpreted as implicit letter-sound correspondence, can also be interpreted as a result of letter-name knowledge. Therefore, future work should empirically investigate letter-sound correspondence in other languages using the same paradigm as our study. Second, it still remains unclear how toddlers acquire implicit letter-sound correspondence. If the implicit letter-sound correspondence can be attributed to environmental exposure but not to a low-level salience effect, non-Japanese speaking toddlers should not show this effect. Future work should investigate this issue by focusing on non-Japanese speaking toddlers. Third, the present study could not examine what kind of letter (e.g., visual complexity) influences this acquisition. Toddlers’ letter-sound correspondence acquisition is preferable to being examined using more items in future studies.

In summary, although toddlers around their third birthday can barely read hiragana, they significantly fixate on sound-matched hiragana letters in the intermodal preferential-looking paradigm. The present results provide new evidence that toddlers possess emergent knowledge of hiragana letter-sound correspondences well before beginning to read the same letters.

## Method

### Participants

A total of 107 Japanese-speaking monolingual toddlers from 24.82 to 47.97 months of age participated in this study. They were recruited from the Keihanna area (Kyoto, Osaka, and Nara border) by advertisements, e-mail, phone, and in person. We excluded 27 toddlers (9 young, 16 middle, and 2 old) due to insufficient eye-tracking data from their head movements and poor oral language development (see data analysis section about detailed exclusion criteria). Our final analysis used 80 toddlers, who were divided into three age groups: young (*N* = 22, *M*_age_ = 28.22 mo., age range = 24.82–31.86 mo., females: 10), middle (*N* = 41, *M*_age_ = 35.89 mo., age range = 32.02–39.98 mo., females: 20), and old (*N* = 17, *M*_age_ = 46.09 mo., age range = 44.68–47.97 mo., females: 10). A previous study showed that Japanese-speaking children at 4;1 and 4;8 years read 46 hiragana letters at mean correct percentages of 25% and 50%^2^, suggesting that they begin to read them from approximately 4 years of age. For oral language development, infants already comprehend the meaning of several words at 6 months^19^ and rapidly increase their productive words from 18 months^30^, indicating that it takes around a year from the comprehension of their first words to the onset of vocabulary explosion. Likewise, we predicated that it takes around a year to read hiragana letters and that children begin to learn implicit hiragana letter-sound correspondence at around 36 months. Therefore, we planned to set 36 months of age at the center of the middle group. Since all groups should have the same age range, we divided the participants into three groups. The present study was approved by the ethics committee of NTT Communication Science Laboratories, and written informed consent was obtained from their parents prior to the experiment. All data were collected in accordance with the relevant guidelines and regulations.

### Materials

Our experiment consisted of 12 trials with six pairs of yoked stimuli:  — (/mi/-/ma/),  — (/ki/-/o/),  — (/shi/-/ko/),  — (/u/-/to/),  — (/ka/-/nu/), and  — (/a/-/ho/) (Table 1). Although testing a large number of stimulus items is statistically critical, a longer experiment complicates toddler participation (e.g., fussiness). This is why we decided the number of items based on a previous study that measured (at 10–18 months) infants’ letter-name and sound knowledge using eye-tracking technique with six items for each measure^23^. The toddlers were presented with a prerecorded sentence that lasted 2.5 s: “X: Which is an X?” Here X stands for the target letters. The sentences were recorded by a Japanese-speaking, 20-year-old female in a sound-treated room.

**Table 1:** Stimuli and letter properties in present study.

	Stimuli	Visual complexity	Reading rank	Stimuli	Visual complexity	Reading rank
Pair 1	/mi/	143.09	2	/ma/	157.74	11
Pair 2	/ki/	159.29	15	/o/	163.31	7
Pair 3	/si/	67.63	4	/ko/	78.19	9
Pair 4	/u/	86.98	10	/to/	90.28	16
Pair 5	/ka/	135.48	1	/nu/	162.16	44
Pair 6	/a/	160.92	12	/ho/	173.06	43

Note that the reading rank is the correct response rate for each hiragana letter in 4- to 5-year-old preschoolers by the National Institute of Japanese Language (1972)^32^.

Since using many items in experiments with young toddlers is difficult, we choose experimental items (letters) to include many types of letters and planned to sample four categories: visual complexity (high vs. low) × acquisition difficulty (high vs. low). However, since the low-complexity and high-difficulty stimuli were quite limited, we only used three categories: (a) high-complexity and low-difficulty, (b) low-complexity and low-difficulty, and (c) high-complexity and high-difficulty (Table 1).

For the visual complexity, we used perimetric complexity, which is nearly proportional with letter identification efficiency^31^. We calculated the perimetric complexity by dividing the squared perimeter by the letter area, i.e., where a higher value implies a visually complex letter. The perimetric complexity for the hiragana characters was calculated based on previous research using an in-house script. The mean value of the hiragana perimetric complexity was 123.79, and we defined visually complex letters (> 135) and visually simple characters (< 95).

For acquisition difficulty, we used the rank of the correct response rate for each hiragana letter in 4- to 5-year-old preschoolers from a previous report^32^. To choose the hiragana letters, we ranked 44 letters of them based on their correct response rates in descending order. The letters within the 16th rank were defined as easy; those above the 28th rank were difficult.

### Apparatus and procedure

We measured the toddlers’ eye movements using Tobii TX300 (Tobii Technology) at 120 Hz. This machine detects eye movements using near-infrared sector lights emitted from the bottom of a 23-in. monitor. The distance from their eyes to the monitor was nearly 60 cm.

First, the experimenter explained the procedure to the parents and obtained written informed consent from them. To verify whether the toddlers could read the letters, we asked their parents to answer the following three statements about their toddlers’ reading abilities of 12 letters: (a) My child does not understand this letter; (b) My child understands this letter but cannot read it; (c) My child can read this letter. However, we omitted item (b) from our analysis because we got no responses for it from the parents. The toddlers participated in the experiment by sitting on their parent’s lap in a testing booth covered on three sides in a dimly lit sound-treated room. The parents wore headphones to prevent them from hearing the target sounds.

**Figure 3:**
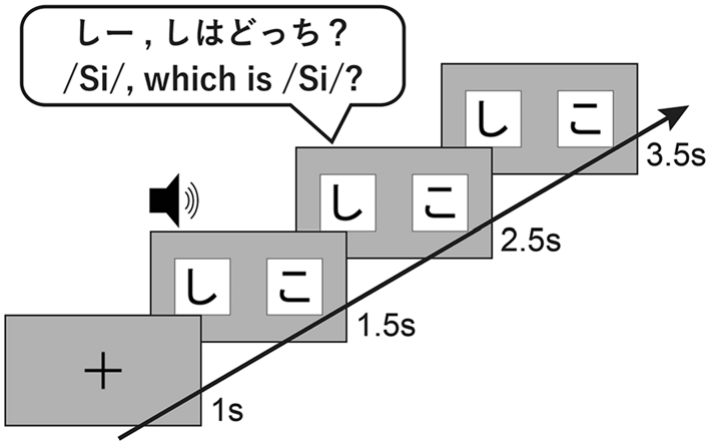
Schematic representation of individual trial procedure. Two hiragana letters were visually presented, and target hiragana sounds were auditorily presented.

In our experiment, six pairs of yoked hiragana stimuli were presented twice, resulting in the evaluation of 12 target hiragana letters (Fig. 3). Before a trial began, a fixation cross was presented at the center of the screen for 1 s, and a short beep was followed by the presentation of the letter pairs. The target hiragana letters were described as “X: Which is an X?” To maintain the toddlers’ attention at the monitor, attention grabbers were presented at the first trial and between trials (i.e., a total of four trials). We moved to the next trial after we verified the toddlers' fixation in the monitor. The side of the target stimuli was counterbalanced to prevent a side bias across participants, and the presentation order of the item-pairs was pseudo-randomized.

After the eye-tracking experiment, parents completed a Japanese version of the MacArthur-Bates Communicative Development Inventories (CDI, word, and grammar)^33^ to assess their vocabulary development (summarized in Table 2). The vocabulary data were obtained from parents whose children were younger than 36-month-olds.

**Table 2:** Summary statistics for CDI score (word production).

Group	Range	Mean	Median
Young (n = 22)	81–653	307.32	259.0
Middle (n = 18)	222–684	479.72	486.5

### Eye-tracking data analysis

First, to exclude the eye-tracking data of the insufficient attention level, this analysis used exclusion criterion and considered more than five trials in which the toddler fixation durations for the target or distractor stimuli were within 500 ms. We also excluded the data from toddlers whose vocabulary level was below the 5th percentile in the CDI. 27 participants were removed from the following analysis.

We evaluated the toddlers’ understanding of letter-sound correspondences using “increase in target looking”^19,20^. This index, which evaluates their word comprehension, was developed to minimize the effect of visual preferences. To calculate it, we measured the fixation duration for the target letter and calculated it by the following formula: the looking ratio at the target letter, for example, ( ) minus its ratio ( ) when it was presented as a distracter. A positive value indicates a preschooler’s fixation for the target stimuli without any visual preference effect. In other words, a positive value is an indicator of an accurate performance of letter-sound correspondences. We evaluated the visual fixation from 367 to 4000 ms after the target sound’s presentation based on previous studies^19,20^. The visual fixation in the area of interest (AOI), defined as the square area surrounding the letters, was counted by the fixation times of the target or distracter stimuli. We calculated the increase in target-looking in a pair-wise manner since the pair was yoked.

## Supplementary information

Supplementary Information.

## Data Availability

The datasets generated during and/or analyzed by the current study are available from the corresponding author on request.
