# Supplementary material for: An eye-tracking study of letter-sound correspondence in Japanese-speaking 2- to 3-year-old toddlers
Source: Sci Rep. 2021 Jan 18;11:1659. doi: 10.1038/s41598-020-79062-y (PMC7814112; doi:10.1038/s41598-020-79062-y)
Supplement: Supplementary file 1 — Supplementary Information. [file 41598_2020_79062_MOESM1_ESM.docx]

**Supplementary information for “An eye-tracking study of letter-sound correspondence in Japanese-speaking 2- to 3-year-old toddlers”**

Hiroki Higuchi, Yuko Okumura, and Tessei Kobayashi

To evaluate which hiragana letters were easily recognized in the eye-tracking task, we calculated the increase in target-looking for each item-pair. We showed this result at the item-pair (not item) level. We choose this analysis because a simple looking ratio for each item might be strongly attributed by visual preference. Since the pair of letters were yoked, we only calculated the ‘increase in target looking’ at the item-pair level. Item-pair analysis revealed that the‘み－ま’ (/mi/-/ma/), ‘か－ぬ’(/ka/-/nu/), and‘し－こ’(/shi/-/ko/) pairs in the old group were significantly larger than zero (p < 0.01, p < 0.01, p = 0.01, respectively, Wilcoxon test). No other item-pair showed significant differences. The‘み–ま’(/mi/–/ma/) and‘か–ぬ’(/ka/–/nu/) pairs were visually complex letters, and the‘し–こ (/si/–/ko/)’pair was visually simple letters, implying that visual complexity is not a strong factor for explaining the performance of the hiragana letter–sound correspondence in the eye-tracking task. Additionally, five of the six letters (*mi*, *ma*, *shi*, *ko*, and *ka*) had high-reading accuracy at 4-6 years of age based on previous data^1^, implying that letters, which are more easily learned as letter-sounds, are also easy for building implicit letter-sounds.


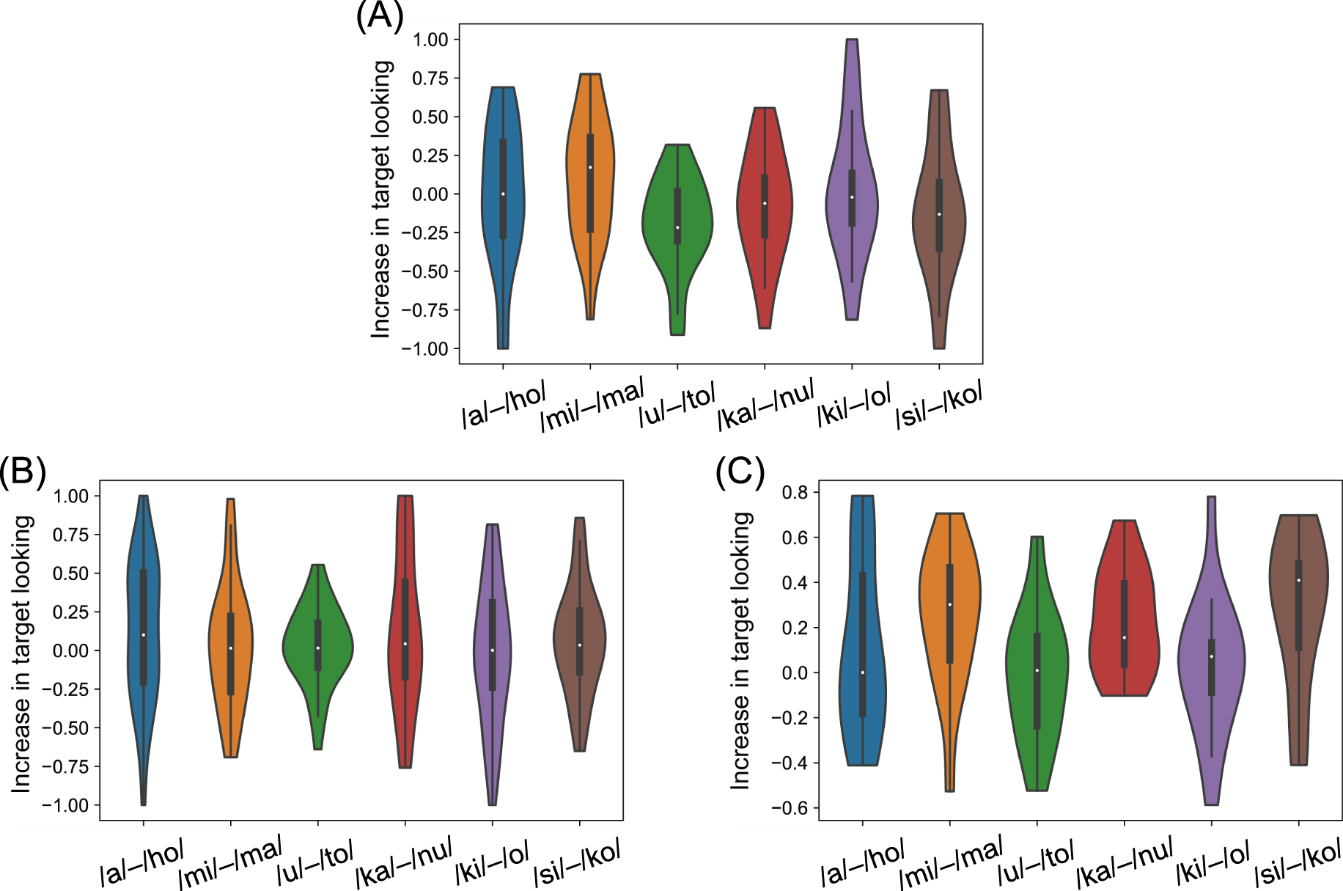


**Supplementary Figure S1** Item-pair means in target-looking increase for three age groups. No significant increase was observed in toddlers in young and middle groups (A, B). ‘み–ま’(/mi/–/ma/),‘か–ぬ’(/ka/–/nu/), and‘し–こ’(/si/–/ko/) pairs in the old group (C) were significantly greater than zero.

**References**

1. National Institute of Japanese Language and Linguistics. *Youji no yomi-kaki nouryoku [Reading and writing ability in preschool children]*. (Tokyo Shoseki, 1972).
